# Anti-Aging Effect of *Agrocybe aegerita* Polysaccharide through Regulation of Oxidative Stress and Gut Microbiota

**DOI:** 10.3390/foods11233783

**Published:** 2022-11-24

**Authors:** Xiaoyan Liu, Linxiu Wu, Aijun Tong, Hongmin Zhen, Dong Han, Hongyang Yuan, Fannian Li, Chengtao Wang, Guangsen Fan

**Affiliations:** 1 Beijing Engineering and Technology Research Center of Food Additives, School of Food and Health, Beijing Technology and Business University, Beijing 100048, China; 2 Engineering Research Centre of Fujian-Taiwan Special Marine Food Processing and Nutrition, Ministry of Education, Fuzhou 350002, China; 3 School of Medicine, South China University of Technology, Guangzhou 510006, China; 4 College of Food Science, Fujian Agriculture and Forestry University, Fuzhou 350002, China

**Keywords:** *Agrocybe aegerita* polysaccharides, anti-aging, antioxidant activity, gut microbiota

## Abstract

Polysaccharides extracted from *Agrocybe aegerita* (AAPS) have various physiological effects. In this study, we used the naturally aging *Drosophila melanogaster* and D-galactose-induced aging mice as animal models to study the anti-aging effects of AAPS via the alleviation of oxidative stress and regulation of gut microbiota. Results showed that AAPS could significantly prolong lifespan and alleviate oxidative stress induced by H_2_O_2_ of *Drosophila melanogaster*. In addition, AAPS significantly increased the activities of antioxidant enzymes in *Drosophila melanogaster* and mice, and reduced the content of MDA. Furthermore, AAPS reshaped the disordered intestinal flora, increased the abundance ratio of *Firmicutes* to *Bacteroidetes*, and increased the abundance of beneficial bacteria *Lactobacillus*. Our results demonstrated that AAPS had good antioxidant and potential anti-aging effects in vivo.

## 1. Introduction

Aging refers to the inevitable degenerative changes of organ functions, eventually leading to disease and death. The aging process is affected by genetic and environmental factors, and the specific aging mechanism is still unclear. The free radical theory is more established in the field of aging research [1]. In 1956, Denham Harman first proposed the free radical theory of aging, arguing that the changes in cellular function during aging are caused by radical oxygen species accumulation, which can lead to biomolecular oxidation and cell damage [2]. Since then, this theory has been widely studied, recognized, and gradually developed into a relatively complete oxidative stress theory. This theory proposes that the synthesis rate and the activity of antioxidant enzymes and substances decline during aging. Thus, the oxidant metabolites accumulate, and the balance between the production and scavenging of free radicals is disrupted, resulting in the accumulation of free radicals. Excess free radicals can trigger lipid peroxidation, destroy the redox homeostasis of cells, cause irreversible tissue damage, and ultimately accelerate the aging process [3,4,5]. Therefore, studying the mechanisms related to aging and anti-oxidation, and exploring natural food with antioxidant activity are essential for addressing aging-related problems.

D-galactose (D-Gal) is a reducing aldohexose. In healthy adults, the galactose from a regular diet can be metabolized into glucose and absorbed by the body. However, long-term and high-dose injections of D-Gal into animals can promote galactose conversion to aldose and hydrogen peroxide under galactose oxidase, which then leads to the accumulation of intracellular reactive oxygen species [6]. The increased reactive oxygen species can cause oxidative stress, inflammation, mitochondrial dysfunction, and apoptosis, all of which contribute to body aging [7]. In addition, *Drosophila melanogaster* has a short life cycle and strong reproductive ability, and it is easy to feed and distinguish genders; moreover, *Drosophila* has very similar pathogenic and aging genes to humans [8]. Therefore, the D-gal-induced aging model in mice and *Drosophila* are widely used to screen antioxidants and anti-aging drugs [9].

*Agrocybe aegerita* is a well-known edible mushroom that grows in northern temperate and subtropical regions, which is popular among people due to its pleasant smell and taste. It has high nutritional value and contains various active ingredients, including polysaccharides, leucine, glutamate and essential amino acid [10]. Accumulating studies show that *A. aegerita* has many physiological functions, e.g., as an antioxidant [11], anti-virus [12], anti-angiogenic [13], and for treating thrombosis [14]. The *A. aegerita* polysaccharide (AAPS) is one of the main active components in its fruit body. Our previous studies have found that AAPS possesses potent anti-aging activity in vitro, representing increased cell viability and decreased mitochondrial membrane potential [15]. It has also been shown to be a suitable prebiotic combined with *lactobacillus rhamnousus* GG alleviating oxidative stress [16]. However, whether AAPS possesses anti-aging effects via regulation of oxidative stress and gut microbiota has not yet been explored in vivo.

In this study, we investigated whether AAPS has an anti-aging effect via alleviating oxidative stress and regulation of gut microbiota. For this purpose, the protective effects of AAPS against natural aging and H_2_O_2_-induced oxidative stress in the *Drosophila* model were evaluated. Moreover, the possibility that AAPS treatments could enhance the antioxidant enzyme activities in *Drosophila* and aging mice was explored. In addition, the gut microbiota in mice was examined by high-throughput sequencing.

## 2. Materials and Methods

### 2.1. Chemicals and Materials

*A. aegerita* (3MTJ-09106) was purchased from Gutian Tianxian Agricultural Products Co., Ltd. (Ningde, China). AAPS was prepared from *A. aegerita* according to our previous works [15]. Lentinan (LE) (Z20080579) was purchased from Hubei Chuangli Pharmaceutical Co., Ltd. (Hubei, China). The kits for estimating total superoxide dismutase (T-SOD) (A001-1-2), total antioxidant capacity (T-AOC) (A015-2-1), glutathione peroxidase (GSH-Px) (A005-1-2), catalase (CAT) (A007-2-1), and malondialdehyde (MDA) (A003-1-1) were purchased from Nanjing Jiancheng Biochemical Co., Ltd. (Nanjing, China). All other chemicals and reagents used were of analytical grade and commercially available.

### 2.2. Fly Strains and Diet

Fly strains used in this experiment were wild-type *Drosophila* melanogaster Oregon (Fujian Agriculture and Forestry University, Fuzhou, China). A basal diet was prepared using the following methods: 31 g of sucrose and 3 g of agar were added to 300 mL of three-distilled water, heated and stirred until fully dissolved. Then, 42 g of corn flour was added to 160 mL of three-distilled water, mixed well, poured into a pot, stirred and boiled until the mixture was pasty. When the mixture was cooled down to room temperature, 3 g of yeast powder and 3 mL of propionic acid were added and stirred. Then 3 mg/mL LE, 3 mg/mL and 9 mg/mL AAPS were added as the experiment medium. The diets were aliquoted into sterilized culture tubes and prepared every three days. Flies were raised in an incubator at (24 ± 1) °C, 50–60% humidity, and 12 h of light/dark cycle.

### 2.3. Lifespan Assay

Lifespan assay was performed as previously described with slight modifications [17]. A total of 800 male and female flies that had not mated within 8 h of eclosion were collected and randomly divided into four groups (200 per group, half male and half female). The normal control group (NC) was fed the basal diet, and the other groups were fed 3 mg/mL Lentinan (LE), 3 mg/mL AAPS (AL) and 9 mg/mL AAPS (AH), respectively. The number of dead fruit flies was recorded at a fixed time every day until all the fruit flies died. The average lifespan and longest lifespan of fruit flies were analyzed. The longest lifespan is the average lifespan of the last 10% of fruit flies in each group.

### 2.4. Hydrogen Peroxide (H_2_O_2_) Challenge Assay

Hydrogen peroxide (H_2_O_2_) challenge assay was performed as previously described with slight modifications [18].H_2_O_2_ was applied to flies to trigger oxidative stress. After 20 days of feeding, a total of 800 male and female flies were collected, and randomly divided into four groups (200 per group, half male and half female), and transferred into new culture tubes containing filter paper saturated with 200 μL of 30% H_2_O_2_ in a 6% glucose solution. The number of dead flies was recorded every three hours until all flies died.

### 2.5. Animals and Treatment

Male Kunming mice, 4–6 weeks, were provided by the Experimental Animal Center of the Fujian Medical University, Fujian, China (quality certificate number: SCXK (Min) 2016-0002). All animals were raised and handled following the Guidelines for the Care and Use of Laboratory Animals. All animal experiments and procedures were approved by the Animal Ethics Committee of Fujian Medical University, and the approval number is 2017-0120.

They were housed at 22 ± 2 °C with a 12 h light-dark cycle and provided free access to food and water. After one week of adaptation, the mice were randomly assigned to 5 groups (*n* = 12): normal control group (NC), model control group with D-Gal (MC), 300 mg/kg.bw/day Lentinan group (LE), 300 mg/kg.bw/day AAPS group (AL), 600 mg/kg.bw/day AAPS group (AH). Except for the control group, all groups were subcutaneously injected with D-galactose dissolved in normal saline (0.9%, *w*/*v*) (120 mg/kg.bw) once daily for 8 weeks. Meanwhile, LE and AAPS groups were orally gavaged with LE or AAPS for 8 weeks; and the NC and MC groups received normal saline solution at the same volume. After the endpoint of the experiment, all animals were sacrificed and the serum, brain, and cecal contents were collected and stored until use.

### 2.6. Biochemical Analysis

Flies from each group were sacrificed by frozen, and homogenized with normal saline in the ice bath. The homogenate was then centrifuged at 3000 r/min for 10 min at 4 °C. The supernatant was collected to measure the protein content, T-SOD activity, CAT activity, GSH-Px activity and MDA content by corresponding kits.

The brain tissues of mice were homogenized and centrifuged, and the supernatant was collected. The supernatant and serum samples were used to measure the protein content, T-AOC activity, CAT activity, GSH-Px activity and MDA content by corresponding kits. All of the procedures completely complied with the manufacturer’s instructions.

### 2.7. Intestinal Microbiota Analysis

Genomic DNA was extracted from mice’s cecal content using the Qiagen DNeasy kit. DNA obtained from each sample was diluted to the same concentration (10 nmol/L) and detected by 1% agarose gel electrophoresis. The V3-V4 region of the bacterial 16S rDNA was amplified using a general primer to generate an amplicon of 480 bp. Illumina MiSeq-PE250 system was employed for the paired-end amplicon sequencing according to manufacturer’s recommendations. For the microbial diversity analysis, the data were analyzed using the Majorbio I-Sanger Cloud Platform (Shanghai, China).

### 2.8. Data Analysis

The experimental results were expressed as mean ± standard deviation (Mean ± SD). GraphPad Prism 8.0 and SPSS 19.0 were used for plotting and statistical processing. Log-rank test was used for the survival test to analyze the significance of life expectancy. D’Agostino & Pearson test was used to assess the normal distribution of data, and then the data was treated in parametric way using Student’s t test. The mean difference was considered significant at *p* < 0.05 or *p* < 0.01.

## 3. Results

### 3.1. Effects of AAPS on the Lifespan of Drosophila

**Figure 1:**
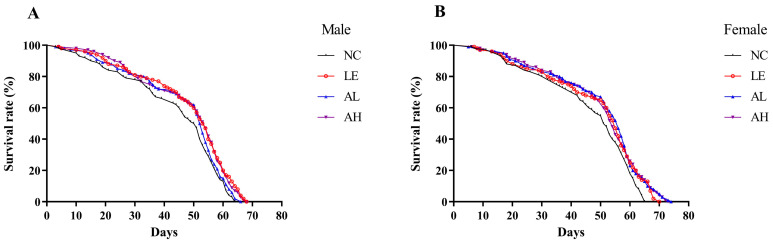
Effects of AAPS on the lifespan of *Drosophila melanogaster.* (**A**) male and (**B**) female. LE, 3 mg/mL Lentinan; AL, 3 mg/mL AAPS; AH, 9 mg/mL AAPS; (*n* = 100 per group).

Aging is a natural process with highly complex progressions. Lifespan is the most direct observational index to evaluate the anti-aging effects in experiments. As shown in Table 1 and Figure 1, AAPS supplementation could extend the lifespan of both female and male fruit flies. Compared with the male NC group, the average lifespan of male fruit flies in the AH group was prolonged from 42.74 to 46.85 days (*p* < 0.05), with a 9.62% increase. The average lifespan of female flies in the AL group was increased from 45.14 to 49.12 days (*p* < 0.05). The longest lifespan of both female and male flies in each AAPS group was significantly prolonged (*p* < 0.05 or *p* < 0.01). Compared with the NC group, the survival curves of AAPS groups were more right dispersed (Figure 1).

### 3.2. Effects of AAPS on H_2_O_2_-Induced Oxidative Stress in Drosophila

**Figure 2:**
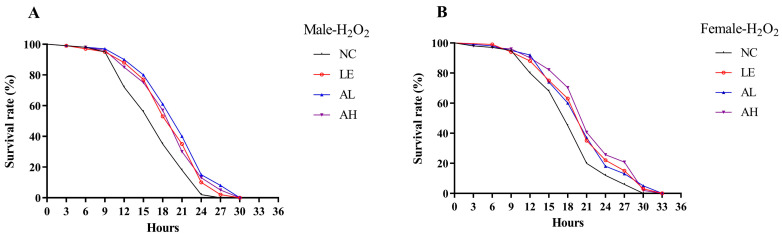
Effects of AAPS on H_2_O_2_ treatment on the survival time of *Drosophila melanogaster.* (**A**) H_2_O_2_ treatment of male (**B**) H_2_O_2_ treatment of female. LE, 3 mg/mL Lentinan; AL, 3 mg/mL AAPS; AH, 9 mg/mL AAPS; (*n* = 100 per group).

Kaplan-Meier survival curve analysis showed AAPS could alleviate oxidative stress caused by H_2_O_2_ (Figure 2). Compared with the male NC group, the average lifespan in AL and AH groups were improved by 19.27% (*p* < 0.01) and 14.41% (*p* < 0.01), respectively (Table 2). The addition of AAPS also extended the lifespan of female groups. As the concentration of AAPS in 3 mg/mL and 9 mg/mL, compared with the control group, the mean lifespans were improved by 11.27% (*p* < 0.01) and 15.94% (*p* < 0.01), respectively. AAPS supplemented also positively extended the longest lifespan of both female and male flies (*p* < 0.01).

### 3.3. Effects of AAPS on the Antioxidant Capacity of Drosophila

**Figure 3:**
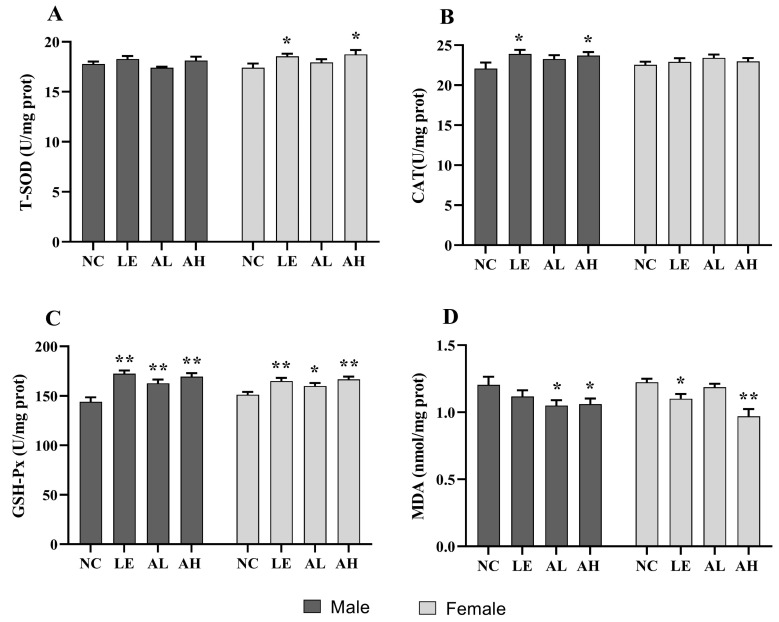
Effects of AAPS on the antioxidant capacity of *Drosophila melanogaster* (*n* = 100 per group). (**A**) T-SOD. (**B**) CAT. (**C**) GSH-Px. (**D**) MDA. LE, 3 mg/mL Lentinan; AL, 3 mg/mL AAPS; AH, 9 mg/mL AAPS. Data are presented as the means ± SEM. * *p* < 0.05 vs. NC group. ** *p* < 0.01 vs. NC group.

After 30 days of AAPS treatment, the content level of MDA and the enzyme activity of T-SOD, CAT, and GSH-Px related to oxidative stress capacity were measured in flies. As shown in Figure 3, the female AH group showed significantly increased T-SOD levels compared with the NC group, with a 7.10% increase (*p* < 0.05). Moreover, the high-AAPS treatment increased the CAT levels in male flies (*p* < 0.05), which was 6.88% higher than the NC group (*p* < 0.05). In addition, the activity of CAT in each AAPS group significantly increased compared with the NC group (*p* < 0.05 or *p* < 0.01). The levels of lipids oxidation end-product MDA in both male and female AH groups were remarkably lower than the NC group (*p* < 0.05).

### 3.4. Effects of AAPS on the Antioxidant Capacity of Mice

**Figure 4:**
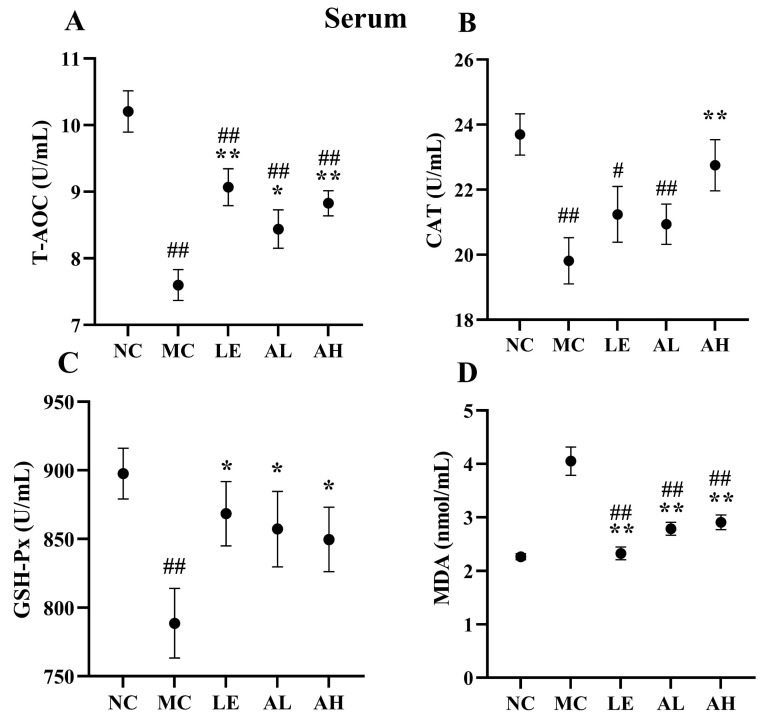
Effects of AAPS on the antioxidant capacity in serum of mice (*n* = 10 per group). (**A**) T-AOC. (**B**) CAT. (**C**) GSH-Px. (**D**) MDA. LE, 300 mg/kg.bw/d Lentinan; AL, 300 mg/kg.bw/d AAPS; AH, 600mg/kg.bw/d AAPS. Data are presented as the means ± SEM. # *p* < 0.05, ## *p* < 0.01 vs. NC group. * *p* < 0.05, ** *p* < 0.01 vs. MC group.

**Figure 5:**
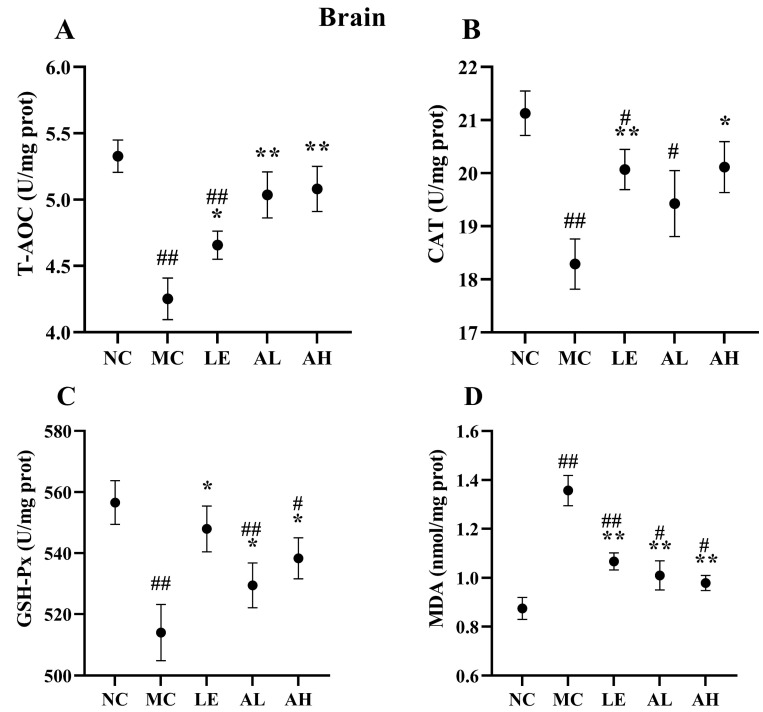
Effects of AAPS on the antioxidant capacity in brain of mice (*n* = 10 per group). (**A**) T-AOC. (**B**) CAT. (**C**) GSH-Px. (**D**) MDA. LE, 300 mg/kg.bw/d Lentinan; AL, 300 mg/kg.bw/d AAPS; AH, 600mg/kg.bw/d AAPS. Data are presented as the means ± SEM. # *p* < 0.05, ## *p* < 0.01 vs. NC group. * *p* < 0.05, ** *p* < 0.01 vs. MC group.

After 8 weeks of AAPS treatment, the AAPS-treated groups showed significant improvement in antioxidant activities (Figure 4 and Figure 5). The antioxidant enzyme (T-AOC, CAT and GSH-Px) activities in serum and brain of the MC group were obviously lower than those of the NC group (all *p* < 0.01). Meanwhile, the antioxidant enzyme (T-AOC, CAT and GSH-Px) activities in the serum and brain of the AH group were significantly increased (*p* < 0.05) than those of the MC group. The level of T-AOC and GSH-Px in the serum and brain of the AL group were also significantly increased (*p* < 0.05). The level of MDA in the serum and brain of two AAPS-treated groups was significantly attenuated (*p* < 0.01). In contrast, those of the MC group were significantly increased compared with the NC group (*p* < 0.01).

### 3.5. Effects of AAPS on the Composition of the Gut Microbiota

**Figure 6:**
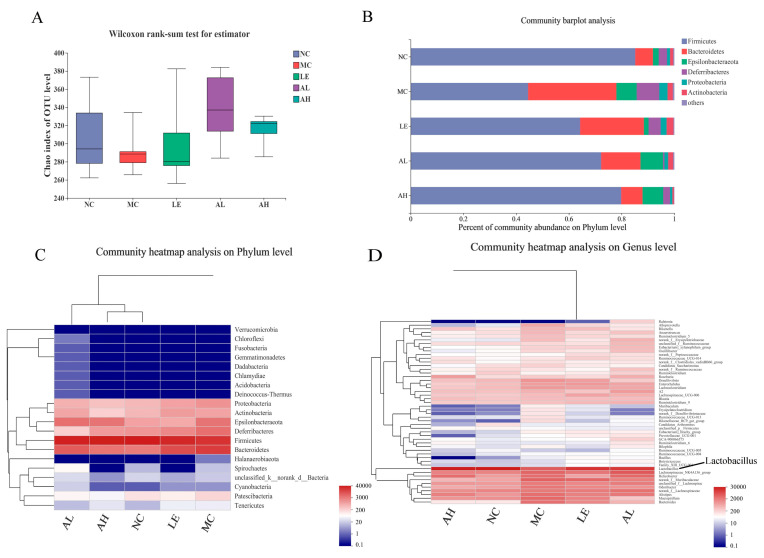
Microbial Alpha diversity and composition analysis of mice treated with different diets. (**A**) Alpha diversity analysis of Chao index of OTU level. (**B**) Relative abundance on the phylum level in barplot analysis. (**C**) Species abundance clustering heatmap on phylum level, the closer distance and the shorter branch length between the two samples suggest that the species composition and abundance of the two samples is more similar; the color gradient from blue to red indicates the relative abundance from low to high. (**D**) Species abundance clustering heatmap on genus level. LE, 300 mg/kg.bw/d Lentinan; AL, 300 mg/kg.bw/d AAPS; AH, 600mg/kg.bw/d AAPS.

Fecal samples were sequenced to assess the effect of AAPS on the gut microbiota structure after 8 weeks of feeding. Then, 16S rDNA raw gene sequences were obtained from 25 samples through Illumina Miseq sequencing. The average number of sequences from each sample was 51,145. As shown in Figure 6A, the average OTU level of the Chao index from AAPS treatment groups was higher than that of the MC group, although there was no significant difference. The community barplot analysis and community heatmap analysis of the microorganism at the phylum level were shown in Figure 6B,C. At the phylum level, the major taxonomic units include *Firmicutes*, *Bacteroidetes*, *Epsilonbacteraeota*, *Deferribacteres* and *Proteobacteria* (Figure 6B). The increased abundance of *Firmicutes* and reduced amount of *Bacteroidetes* and *Proteobacteria* in the AAPS treatment groups indicated an increase in beneficial bacteria and a reduction in detrimental bacteria, compared with the MC group. The ratio of *Firmicutes* to *Bacteroidetes* was also increased. By displaying the major phylotypes in the heatmap, we observed that samples of AAPS treatment groups were clustered with that of the NC group (Figure 6C), which indicated that AAPS reshaped the disordered intestinal flora induced by D-gal injection. The abundances of beneficial *Lactobacillus* in the intestine of the AAPS treatment groups were higher than that of the MC group (Figure 6D).

**Figure 7:**
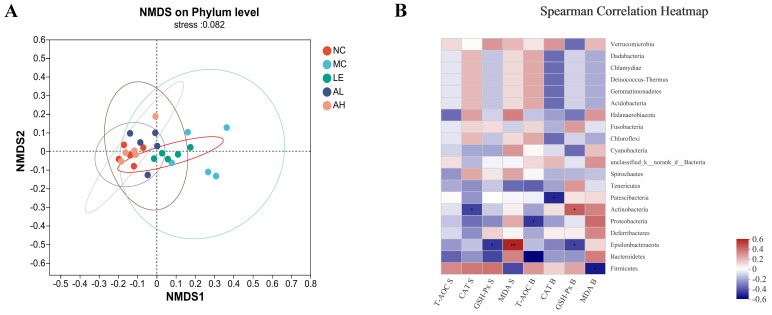
Beta diversity analysis of gut microbiota and correlation analysis between the relative abundance of gut microbiota and biomarkers. (**A**) Non-metric Multidimensional Scaling (NMDS) analysis on phylum level based on Bray-Curtis metrics. (**B**) Spearman correlation heatmap analysis between the relative abundance of gut microbiota and the oxidative stress biomarkers. LE, 300 mg/kg.bw/d Lentinan; AL, 300 mg/kg.bw/d AAPS; AH, 600 mg/kg.bw/d AAPS. * *p* < 0.05 vs. NC group. ** *p* < 0.01 vs. NC group.

The similarity of the microbial communities in the five groups of mice was analyzed by Non-metric Multidimensional Scaling (NMDS), based on Bray-Curtis distance metrics (Figure 7A). The scatter plot showed that the microbiota of the NC group and AAPS treatment groups were mixed, while the MC group was different from other groups. In addition, the Spearman correlation heatmap showed the potential correlations between the oxidative stress indicators and gut microbiota (Figure 7B). *Epsilonbacteraeota* exhibited a positive with the MDA level in the serum, and was negatively associated with the GSH-Px level in both serum and brain. *Patescibacteria* exhibited a negative correlation with the CAT lever in the brain. *Actinobacteria* abundance was positively correlated with the GSH-Px levels in brain, but was negatively correlated with the expression level of CAT in serum. Furthermore, *Proteobacteria* and *Bacteroidetes* exhibited a negative correlation with the T-AOC level in the brain, while *Firmicutes* was negatively correlated with the MDA level in the brain.

## 4. Discussion

Aging is the gradual decline of body’s physiological integrity and adaptability over time, resulting in the loss of tissue functions [19]. There are many theories about aging, but the mechanisms of its occurrence and development have not been fully elucidated. Among the proposed theories, oxidative stress, inflammatory aging, and intestinal microbiota imbalance are the relatively established theories of aging [20,21,22]. In this study, the AAPS supplement enhanced the antioxidant capacity of D-galactose-induced aging mice and changed the structure of intestinal microbiota; moreover, the increase in antioxidant levels was strongly correlated with the changes in the abundance of critical intestinal bacteria. During aging, the activity of free radical scavenging enzymes is reduced. The ability to scavenge free radicals is weakened, resulting in a considerable accumulation of free radicals, which in turn causes DNA mutation, protein function damage, cell structure and organelle function damage, and ultimately accelerates aging [23]. Therefore, improving the activity of antioxidant enzymes and reducing oxidative stress can delay aging.

The antioxidant system in the body, including the glutathione system and superoxidase, is an essential part of maintaining the intracellular redox balance [24,25]. Thus, the degree of aging can be indicated by oxidative stress biomarkers. Total antioxidants are the sum of the body’s non-enzymatic and enzymatic system antioxidants, and T-AOC can comprehensively reflect the body’s antioxidant capacity. CAT is another antioxidant enzyme in the body, which can scavenge free radicals, thereby reducing the peroxide produced by the body [26]. MDA is the final product of lipid peroxidation induced by free radicals on unsaturated fatty acids. It is cross-linked with proteins, peptides, or lipid polymers to continuously accumulate senile pigments or lipofuscin, resulting in body aging [27,28]. Therefore, MDA content can objectively reflect free radicals’ level and lipid peroxidation degree. Our study showed that the effect of AAPS on the lifespan of Drosophila was related to the increase of antioxidant enzyme activity and the decrease of MDA concentration, suggesting a causal relationship between antioxidant enzyme activity and aging (Figure 3). In addition, the MDA level of the aging mice treated with D-galactose was higher than that of the NC group, and others antioxidant enzyme level were lower, indicating that the aging body was in a state of oxidative stress. After supplementing with different concentrations of AAPS, the oxidative stress was significantly alleviated (Figure 4 and Figure 5). Previous studies have reported the antioxidant effects of AAPS, including the strong ability of free radicals scavenging in vitro and improving the activity of antioxidant enzymes in the body, which are consistent with the results of our study [11,15,16]. However, further research is needed to reveal the molecular mechanism of AAPS increasing the activity of antioxidant enzymes and delaying aging.

Although the structure of intestinal microbiota is affected by various factors such as country, region, dietary habits, gender, and disease, it is still considered to be one of the critical factors affecting longevity. Many studies have found that changes in the composition of the intestinal microbiota are closely related to host aging [29,30,31]. The diversity of microbiota is positively correlated with body health and negatively correlated with gut vulnerability in the elderly [32]. Biagi et al. and Kong et al. found that the extremely long-lived people in China and Italy had greater intestinal microbiota diversity than young adults [32,33]. In this study, the Alpha diversity results showed that the average OTU level of the Chao index from AAPS treatment groups was higher than that of the MC group, although there was no significant difference (Figure 6A).

The intestinal microbiota structure of the elderly is very different from that of the young people, with decreased abundance of *Firmicutes* and an increased abundance of *Baceteroidetes* [34]. Sagi et al. found that, compared with wild-type (SOD1^+/+^) mice, the mice lacking antioxidant enzyme SOD1 (SOD1^−/−^) exhibited altered ratios of *Firmicutes* to *Bacteroidetes*; moreover, the redox imbalance caused by SOD1 deletion also altered intestinal microbiota and its metabolites [35]. The ratio of *Firmicutes* to *Bacteroidetes* decreased significantly in the elderly, which is consistent with the aging model group in our study. Notably, AAPS also significantly increased the ratio (Figure 6B).

*Lactobacilli* are known as health-promoting bacteria, which have long been regarded as one of the most abundant microorganisms in the human gastrointestinal tract. They are also recognized as beneficial host-related groups in human and animal microbiota [36,37]. Li et al. [38] not only confirmed that *Lactobacillus* had good free radical scavenging ability in vitro, but also found that administration of *Lactobacillus* in vivo significantly improved the aging-related indicators, and reduced the D-galactose-induced hepatic oxidative stress by modulating the Nrf-2 signaling pathway. Redox imbalance in the body can cause oxidative damage and alter the intestinal microbiota. In our study, AAPS treatment had a more significant impact on the relative abundance of *Lactobacillus* compared with the MC group (Figure 6D). The abundance of *Lactobacilli* in the intestine of the MC group was lower than that of other groups, indicating that the aging body is in a state of microbiota imbalance. After intervening with different concentrations of AAPS, the serum MDA of the mice was significantly decreased and the antioxidant enzyme activities were significantly increased; in addition, the abundance of beneficial *Lactobacilli* in the intestine was increased. This result is consistent with the findings from Kong et al. [39], and the increase in the composition and abundance of *Lactobacillus* helps to relieve oxidative stress. Therefore, our results suggest that AAPS may exhibit antioxidant effects by regulating the composition and abundance of *Lactobacillus* in the intestinal microbiota of the aging body.

Furthermore, the correlation study on anti-aging indicators after AAPS treatment and dominant bacteria in intestinal microbiota found that, *Epsilonbacteraeota* exhibited a positive with MDA level in serum, and was negatively associated with the GSH-Px level in both serum and brain. Moreover, *Proteobacteria* and *Bacteroidetes* exhibited a negative correlation with the T-AOC level in the brain. At the same time, *Firmicutes* was negatively correlated with the MDA level in the brain (Figure 7B). This result further demonstrated that AAPS might regulate the structure and abundance of intestinal microbiota, which further affects the oxidative stress of the body and delays aging.

## 5. Conclusions

Population aging is a significant issue faced by all countries in the world. During aging, the body’s physiological integrity and adaptability gradually decline, resulting in a gradual loss of tissue functions. Therefore, the enhancement of anti-oxidative stress function and the improvement of intestinal microbiota has become one of the bases for anti-aging strategies. This study confirmed that *Agrocybe aegerita* polysaccharide could prolong the lifespan of *Drosophila melanogaster*, alleviate oxidative stress induced by H_2_O_2_, enhance the anti-oxidative stress ability of *Drosophila* and mice, and regulate the composition of intestinal microbiota in mice, suggesting that AAPS has good antioxidant and anti-aging functions. However, this study has not yet fully elucidated the mechanism of how changes in intestinal microbiota mediated by AAPS treatment regulate oxidative stress and aging-related signaling pathways. Therefore, it is necessary to further investigate the roles of intestinal microbiota and its metabolites in the antioxidant and anti-aging functions of AAPS from the perspective of molecular biology, which will help to reveal the anti-aging mechanism of AAPS comprehensively.

## Figures and Tables

**Table 1:** Effects of *Agrocybe aegerita* polysaccharide on the lifespan of *Drosophila*.

Group		Mean Lifespan (d)	Maximum Lifespan (d)	Median Survival (d)	Prolongation of Mean Life Span (%)
Male	NC	42.74 ± 1.66	61.70 ± 0.38	50.5	-
	LE	47.00 ± 1.60 *	65.40 ± 0.32 **	53.5	9.97
	AL	45.57 ± 1.56	62.80 ± 0.42 *	52.5	6.62
	AH	46.85 ± 1.53 *	64.60 ± 0.47 **	54	9.62
Female	NC	45.14 ± 1.61	63.20 ± 0.24	51.5	-
	LE	47.91 ± 1.67	67.10 ± 0.33 **	55	6.14
	AL	49.12 ± 1.64 *	69.70 ± 0.66 **	56	8.82
	AH	48.79 ± 1.61	69.00 ± 0.60 **	54	8.09

Note: data are presented as the means ± SEM (*n* = 100 per group). * *p* < 0.05 vs. NC group. ** *p* < 0.01 vs. NC group.

**Table 2:** Effects of *Agrocybe aegerita* polysaccharide on the lifespan of H_2_O_2_ treated *Drosophila*.

Group		Mean Lifespan (h)	Maximum Lifespan (h)	Median Survival (h)	Prolongation of Mean Life Span (%)
Male	NC	17.28 ± 0.48	24.60 ± 0.38	18	-
	LE	19.68 ± 0.52 **	27.60 ± 0.38 **	21	13.89
	AL	20.61 ± 0.53 **	29.10 ± 0.43 **	21	19.27
	AH	19.77 ± 0.53 **	28.50 ± 0.47 **	21	14.41
Female	NC	18.63 ± 0.57	28.80 ± 0.46	18	-
	LE	20.82 ± 0.61 **	30.90 ± 0.43 **	21	11.76
	AL	20.73 ± 0.61 **	31.50 ± 0.47 **	21	11.27
	AH	21.60 ± 0.63 **	30.60 ± 0.38 **	21	15.94

Note: data are presented as the means ± SEM (*n* = 100 per group). ** *p* < 0.01 vs. NC group.

## Data Availability

All data have been provided in this manuscript.

## References

[1] Martemucci G., Portincasa P., Ciaula A.D., Mariano M., Centonze V., D’Alessandro A.G. (2022). Oxidative stress, aging, antioxidant supplementation and their impact on human health: An overview. Mech. Ageing Dev..

[2] Denham H. (1956). Aging: A theory based on free radical and radiation chemistry. J. Gerontol..

[3] Wojtunik-Kulesza K.A., Oniszczuk A., Oniszczuk T., Waksmundzka-Hajnos M. (2016). The influence of common free radicals and antioxidants on development of Alzheimer’s disease. Biomed. Pharmacother..

[4] Forman H.J. (2016). Redox signaling: An evolution from free radicals to aging. Free Radic. Biol. Med..

[5] Ionescu-Tucker A., Cotman C.W. (2021). Emerging roles of oxidative stress in brain aging and Alzheimer’s disease. Neurobiol. Aging..

[6] Azman K.F., Zakaria R. (2019). D-Galactose-induced accelerated aging model: An overview. Biogerontology.

[7] Shwe T., Pratchayasakul W., Chattipakorn N., Chattipakorn S.C. (2018). Role of D-galactose-induced brain aging and its potential used for therapeutic interventions. Exp. Gerontol..

[8] Lee S.H., Min K.J. (2019). *Drosophila melanogaster* as a model system in the study of pharmacological interventions in aging. Transl. Med. Aging..

[9] Taormina G., Ferrante F., Vieni S., Grassi N., Russo A., Mirisola M.G. (2019). Longevity: Lesson from model organisms. Genes.

[10] Lee K.J., Yun I.J., Kim K.H., Lim S.H., Ham H.J., Eum W.S., Joo J.H. (2011). Amino acid and fatty acid compositions of *Agrocybe chaxingu*, an edible mushroom. J. Food Compos. Anal..

[11] Jing H., Li J., Zhang J., Wang W., Li S., Ren Z., Gao Z., Song X., Wang X., Le J. (2018). The antioxidative and anti-aging effects of acidic- and alkalic-extractable mycelium polysaccharides by *Agrocybe aegerita* (Brig.) Sing. Int. J. Biol. Macromol..

[12] El-Maradny Y.A., El-Fakharany E.M., Abu-Serie M.M., Hashish M.H., Selim H.S. (2021). Lectins purified from medicinal and edible mushrooms: Insights into their antiviral activity against pathogenic viruses. Int. J. Biol. Macromol..

[13] Lin S., Ching L.T., Lam K., Cheung P.C.K. (2017). Anti-angiogenic effect of water extract from the fruiting body of *Agrocybe aegerita*. LWT Food Sci. Technol..

[14] Li G., Liu X., Cong S., Deng Y., Zheng X. (2021). A novel serine protease with anticoagulant and fibrinolytic activities from the fruiting bodies of mushroom *Agrocybe aegerita*. Int. J. Biol. Macromol..

[15] Liu X., Liu D., Chen Y., Zhong R., Gao L., Yang C., Ai C., El-Seedi H.R., Zhao C. (2020). Physicochemical characterization of a polysaccharide from *Agrocybe aegirita* and its anti-ageing activity. Carbohydr. Polym..

[16] Wu L., Liu X., Hu R., Chen Y., Xiao M., Liu B., Zeng F. (2022). Prebiotic *Agrocybe cylindracea* crude polysaccharides combined with *Lactobacillus rhamnosus GG* postpone aging-related oxidative stress in mice. Food Funct..

[17] Zhang J., Liu X., Pan J., Zhao Q., Li Y., Gao W., Zhang Z. (2020). Anti-aging effect of brown black wolfberry on *Drosophila melanogaster* and d-galactose-induced aging mice. J. Funct. Foods.

[18] Cai X., Chen S., Liang J., Tang M., Wang S. (2021). Protective effects of crimson snapper scales peptides against oxidative stress on *Drosophila melanogaster* and the action mechanism. Food Chem. Toxicol..

[19] Kemoun P.H., Ader I., Planat-Benard V., Dray C., Fazilleau N., Monsarrat P., Cousin B., Paupert J., Ousset M., Lorsignol A. (2022). A gerophysiology perspective on healthy ageing. Ageing Res. Rev..

[20] Conway J., Duggal N.A. (2021). Ageing of the gut microbiome: Potential influences on immune senescence and inflammageing. Ageing Res. Rev..

[21] Barone M., D’Amico F., Rampelli S., Brigidi P., Turroni S. (2022). Age-related diseases, therapies and gut microbiome: A new frontier for healthy aging. Mech. Ageing Dev..

[22] Travier L., Singh R., Fernández D.S., Deczkowska A. (2022). Microbial and immune factors regulate brain maintenance and aging. Curr. Opin. Neurobiol..

[23] Warraich U.A., Hussain F., Kayani H.R. (2020). Aging—Oxidative stress, antioxidants and computational modeling. Heliyon.

[24] Demirci-Çekiç S., Özkan G., Avan A.N., Uzunboy S., Çapanoğlu E., Apak R. (2022). Biomarkers of oxidative stress and antioxidant defense. J. Pharm. Biomed..

[25] Pisoschi A.M., Pop A., Iordache F., Stanca L., Predoi G., Serban A.I. (2021). Oxidative stress mitigation by antioxidants—An overview on their chemistry and influences on health status. Eur. J. Med. Chem..

[26] Nandi A., Yan L.J., Jana C.K., Das N. (2019). Role of catalase in oxidative stress-and age-associated degenerative diseases. Oxid. Med. Cell Longev..

[27] Mas-Bargues C., Escrivá C., Dromant M., Borrás C., Viña J. (2021). Lipid peroxidation as measured by chromatographic determination of malondialdehyde. Human plasma reference values in health and disease. Free Radic. Biol. Med..

[28] Tsikas D. (2017). Assessment of lipid peroxidation by measuring malondialdehyde (MDA) and relatives in biological samples: Analytical and biological challenges. Anal. Biochem..

[29] Vaiserman A.M., Koliada A.K., Marotta F. (2017). Gut microbiota: A player in aging and a target for anti-aging intervention. Ageing Res. Rev..

[30] Coman V., Vodnar D.C. (2020). Gut microbiota and old age: Modulating factors and interventions for healthy longevity. Exp. Gerontol..

[31] Tiihonen K., Ouwehand A.C., Rautonen N. (2010). Human intestinal microbiota and healthy ageing. Ageing Res. Rev..

[32] Biagi E., Franceschi C., Rampelli S., Severgnini M., Ostan R., Turroni S., Consolandi C., Quercia S., Scurti M., Monti D. (2016). Gut microbiota and extreme longevity. Curr. Biol..

[33] Kong F., Hua Y., Zeng B., Ning R., Li Y., Zhao J. (2016). Gut microbiota signatures of longevity. Curr. Biol..

[34] Kumar M., Babaei P., Ji B., Nielsen J. (2016). Human gut microbiota and healthy aging: Recent developments and future prospective. Nutr. Health Aging.

[35] Sagi H., Shibuya S., Kato T., Nakanishi Y., Tsuboi A., Moriya S., Ohno H., Miyamoto H., Kodama H., Shimizu T. (2020). SOD1 deficiency alters gastrointestinal microbiota and metabolites in mice. Exp. Gerontol..

[36] Li Q., Gänzle M.G. (2020). Host-adapted *lactobacilli* in food fermentations: Impact of metabolic traits of host adapted *lactobacilli* on food quality and human health. Curr. Opin. Food Sci..

[37] Remus D.M., Kleerebezem M., Bron P.A. (2011). An intimate tête-à-tête—How probiotic *lactobacilli* communicate with the host. Eur. J. Pharmacol..

[38] Li B., Evivie S.E., Lu J., Jiao Y., Wang C., Li Z., Liu F., Huo G. (2018). *Lactobacillus helveticus* KLDS1.8701 alleviates d-galactose-induced aging by regulating Nrf-2 and gut microbiota in mice. Food Funct..

[39] Kong Y., Olejar K.J., On S.L.W., Chelikani V. (2020). The potential of *lactobacillus* spp. for modulating oxidative stress in the gastrointestinal tract. Antioxidants.

